# An overview of hyperbaric oxygen preconditioning against ischemic stroke

**DOI:** 10.1007/s11011-023-01165-y

**Published:** 2023-02-02

**Authors:** Xuyi Wu, Jiuhong You, Xinxin Chen, Mei Zhou, Hui Ma, Tianle Zhang, Cheng Huang

**Affiliations:** 1 Rehabilitation Medicine Center, West China Hospital, Sichuan University, Chengdu, Sichuan China; 2 Key Laboratory of Rehabilitation Medicine in Sichuan Province, West China Hospital, Sichuan University, Chengdu, Sichuan China; 3 School of Rehabilitation Sciences, West China School of Medicine, Sichuan University, Chengdu, Sichuan China; 4 West China School of Nursing, Sichuan University, Chengdu, Sichuan China; 5 Department of Neurosurgery, West China Hospital, Sichuan University, Chengdu, Sichuan China

**Keywords:** Hyperbaric oxygen therapy, Preconditioning, Ischemic stroke, Antioxidant capacity, Stem cell, Hypoxia-inducible factor-1

## Abstract

Ischemic stroke (IS) has become the second leading cause of morbidity and mortality worldwide, and the prevention of IS should be given high priority. Recent studies have indicated that hyperbaric oxygen preconditioning (HBO-PC) may be a protective nonpharmacological method, but its underlying mechanisms remain poorly defined. This study comprehensively reviewed the pathophysiology of IS and revealed the underlying mechanism of HBO-PC in protection against IS. The preventive effects of HBO-PC against IS may include inducing antioxidant, anti-inflammation, and anti-apoptosis capacity; activating autophagy and immune responses; upregulating heat shock proteins, hypoxia-inducible factor-1, and erythropoietin; and exerting protective effects upon the blood-brain barrier. In addition, HBO-PC may be considered a safe and effective method to prevent IS in combination with stem cell therapy. Although the benefits of HBO-PC on IS have been widely observed in recent research, the implementation of this technique is still controversial due to regimen differences. Transferring the results to clinical application needs to be taken carefully, and screening for the optimal regimen would be a daunting task. In addition, whether we should prescribe an individualized preconditioning regimen to each stroke patient needs further exploration.

## Introduction

As the population ages, ischemic stroke (IS) has become the second leading cause of morbidity and mortality in the world, causing temporary or permanent physical, cognitive, behavioral, and emotional disorders in adults. The pathological mechanisms of IS are complex and are usually characterized by hypoxia, oxidative stress, inflammation, microvascular dysfunction, apoptosis, necrosis, and ultimately cell death. Recently, with the advancement of revascularization procedures and recanalization of the occluded cerebral arteries, significant improvements have been made in reducing brain damage after IS. Although the mortality from IS has decreased, the incidence of dysfunction has increased. There are still limited effective methods to improve function in clinical practice. Growing evidence from developed countries suggests that prevention should be given high priority to reduce the burden of stroke (Katan and Luft 2018).

Over the past few decades, the search for new strategies that can protect neural cells from ischemia has made “ischemic preconditioning” the most effective method of endogenous protection. Since Kitagawa et al. first reported that neurocytes became resistant to subsequent fatal ischemia after a brief period of subfatal ischemic preconditioning in 1990 (Kitagawa et al. 1990), cerebral ischemic tolerance has been extensively studied. However, despite its promise, ischemic preconditioning is usually invasive and difficult to control in clinical practice, making it difficult to achieve an overall net positive effect in improving postischemic function. In addition to hypoxia, many preconditioning stimuli have also been investigated in the laboratory, such as hypo- and hyperthermia, heavy metals, ethanol, neurotoxins, and other drugs. The phenomenon of “cross-tolerance”(Stetler et al. 2014), in which sublethal stimuli protect against different injuries, proposed that different preconditioning stimuli might protect against a variety of injuries. At present, it is unclear whether these methods are harmful to humans or truly clinically beneficial. Hyperbaric oxygen (HBO) therapy is performed in a pressurized chamber at 100% oxygen at a pressure above normal atmospheric pressure. It is an effective method to increase the arterial oxygen partial pressure and oxygen supply by increasing oxygen dissolved in the plasma, thus promoting cell metabolism and maintaining adenosine-triphosphate synthesis in injured tissues. Hyperbaric oxygen preconditioning (HBO-PC) refers to exposure to HBO prior to a critical event, with the plan to create a prophylactic treatment situation. HBO-PC is a well-known protective nonpharmacological preconditioning method in patients with IS with encouraging results (Camporesi and Bosco 2014; Francis and Baynosa 2017), but the mechanisms of action have not yet been ascertained.

In this paper, we concisely reviewed the pathophysiology of IS and revealed the underlying mechanism of HBO-PC in protection against IS.

## HBO preconditioning

Preconditioning is a phenomenon in which transient sublethal insult induces robust protection against subsequent lethal injuries. It was first discovered in the heart (Murry et al. 1986) and then shown to occur in the brain and other organisms. Effective preconditioning stimuli vary from transient ischemia, hypoxia, HBO, and low and high temperatures to exposure to neurotoxins and pharmacological agents (Stetler et al. 2014). Although heavy metals, toxins, ethanol, and ionizing radiation have been successfully used to induce ischemic tolerance in animal models (Fang et al. 2020; Kokošová et al. 2014), they are not safe for clinical practice in patients. The goal of developing a safe, practical approach for chemical tolerance seems paradoxical until oxygen is considered.

HBO is often used to treat patients with decompression sickness, carbon monoxide poisoning, and arterial gas embolism and is used as an adjunct treatment for a variety of diseases with injured oxygen delivery (Sheridan and Shank 1999; Tibbles and Edelsberg 1996). In addition, over the past 30 years, studies have shown that HBO can induce ischemic tolerance without harmful side effects. A study indicated that single HBO session at two atmospheres for an hour at a time can induce brain tolerance to ischemic neuronal injury, in which the induction of heat shock protein 72 is crucial (McLaughlin et al. 2003; Wada et al. 1996). Subsequently, the protective effects of HBO pretreatment have been studied in a variety of animals in addition to stroke models (Prass et al. 2000; Wada et al. 1996; Xiong et al. 2000), such as surgical brain injury (Jadhav et al. 2009), ischemia–reperfusion injury(Wang et al. 2021), neonatal hypoxia-ischemia(Li et al. 2008c), intracerebral hemorrhage (Wang et al. 2019), spinal cord ischemia (Hirata et al. 2007), cardiac ischemia (Kim et al. 2001), and liver dysfunction (Yu et al. 2005). In addition, a recent study reported that single-dose HBO pretreatment provides a neuroprotective effect similar to that of hypoxic pretreatment in neonatal mice, while HBO is much safer than other stimuli, such as hypoxia (Freiberger et al. 2006). However, the underlying mechanisms of its neuroprotective effect remain poorly defined.

## Potential mechanism of HBO-PC for IS

**Fig. 1:**
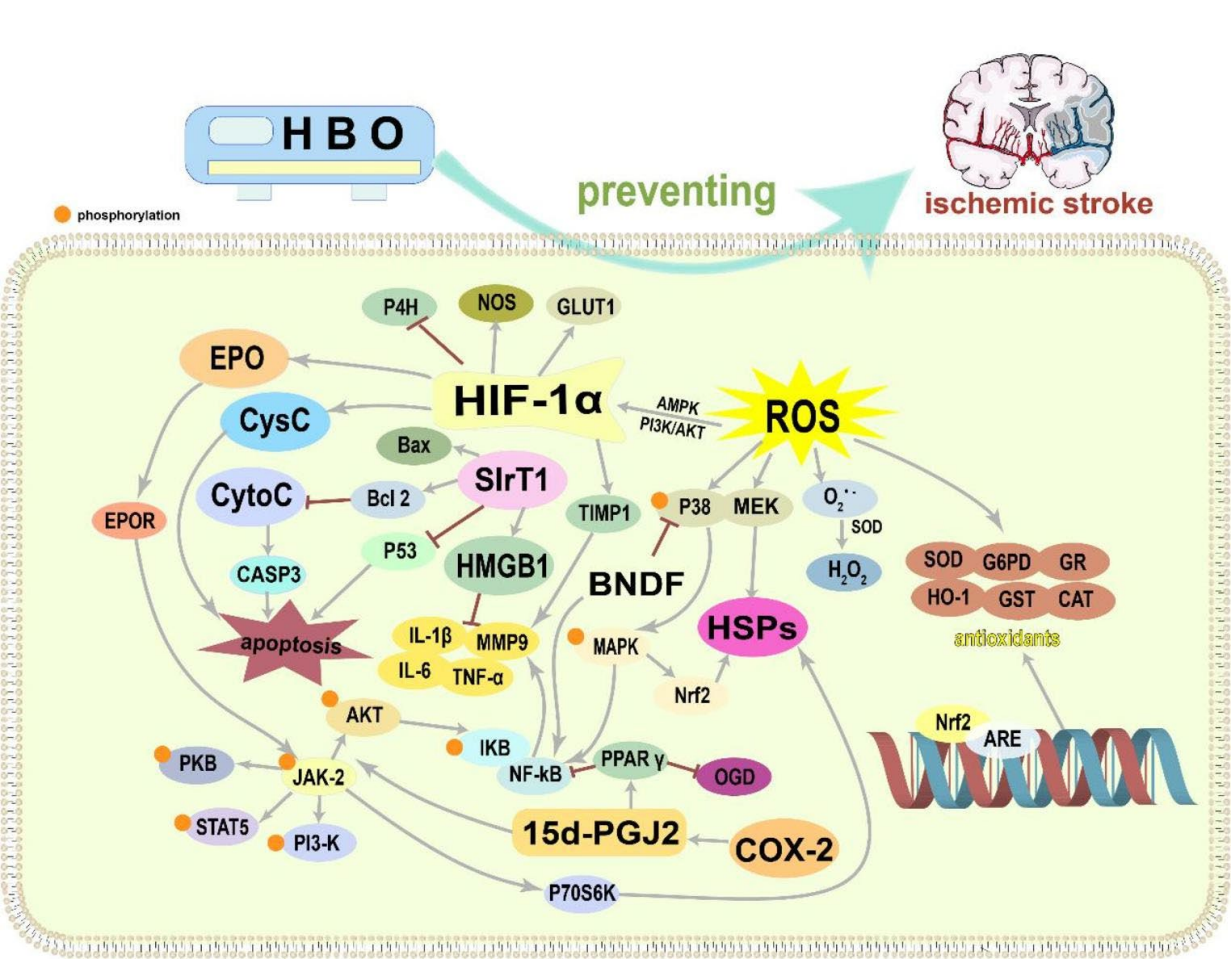
Potential mechanism of hyperbaric oxygen preconditioning for ischemic stroke. Abbreviations: HBO, hyperbaric oxygen; HIF-1α, hypoxia-inducible factor-1α; EPO, erythropoietin; EPOR, erythropoietin receptor; P4H, prolyl 4-hydroxylase; NOS, nitric oxide synthase; GLUT1, glucose transporter 1; CysC, Cystatin C; TIMP-1, tissue inhibitor of metalloproteinases-1; IL-6, interleukin-6; IL-1β, interleukin-1β; MMP9, matrix metalloproteinase-9; TNF-α, tumor necrosis factor-α; SIRT1, Sirtuin 1; HMGB1, High mobility group box 1; CytoC, Cytochrome C; CASP3, caspase3; ROS, reactive oxygen species; AMPK, AMP-activated protein kinase; PI3K/AKT, phosphoinositide 3-kinase/Akt; BDNF, brain-derived neurotrophic factor; NF-κB, nuclear factor kappa-B; MAPK, mitogen-activated protein kinase; HSPs, heat shock proteins; Nrf2, NF-E2-related factor 2; PPAR γ, peroxisome proliferator-activated receptors γ; OGD, oxygen-glucose deprivation; 15d-PGJ2, 15- deoxy-Δ12,14-PGJ2; COX-2, Cyclooxygenase-2; p70 S6 K, ribosomal protein S6 kinase; JAK-2, Janus tyrosine kinase-2; PKB, protein kinase B; STAT5, Signal transducer and activator of transcription 5; PI3-K, phosphatidylinositol-3-kinase; H_2_O_2_, hydrogen peroxide; SOD, superoxide dismutase; G6PD, glucose-6-phosphate dehydrogenase; HO-1, heme oxygenase-1; GST, glutathione-S transferase; CAT, Catalase; ARE, antioxidant response element.

IS refers to the occurrence of ischemic/hypoxic injury in the brain area that blocks the blood supply of arteries due to sudden occlusion of cerebral vessels, and eventually the formation of ischemic core and ischemic penumbra (Patil et al. 2022). The potential mechanisms of the preventive effects of HBO-PC against IS may include nine aspects: (1) induction of antioxidant capacity; (2) generation of hypoxia-inducible factor-1α (HIF-1α) and erythropoietin (EPO); (3) acceleration of heat shock protein (HSP) expression; (4) reduction of inflammation and regulation of immune function; (5) activation of autophagy; (6) inhibition of apoptosis; (7) neuroprotection via brain-derived neurotrophic factor (BDNF); (8) protection of the integrity of the blood–brain barrier (BBB) and reduction of matrix metalloproteinase-9 (MMP-9); and (9) combination with stem cell therapy to prevent IS. The potential mechanism of HBO-PC for IS is shown in Fig. 1.

### HBO-PC and induction of antioxidant capacity

#### Production of ROS

ROS are produced in various ways. The primary source of ROS is mitochondrial respiratory chain activity and NADPH oxidase (NOX) (Adam-Vizi 2005). Under normal conditions, a small fraction of the electrons steaming across the electron delivery chain react with O_2_ to constitute superoxide anion (O_2_^−^), a highly reactive oxidant that is transformed by superoxide dismutase (SOD) to a less toxic molecule, hydrogen peroxide (H_2_O_2_). Then, it is further transformed to H_2_O and O_2_ by catalase (CAT) or to H_2_O by glutathione peroxidase (GPx). It can also transfer its electron to produce other oxidants peroxynitrite (ONOO^−^). There are other antioxidants that transform superoxide to oxygen, such as lactoferrin (an iron-binding protein).

It is easy to understand that HBO increases ROS production (Benedetti et al. 2004; de Wolde et al. 2022; Gasier and Fothergill 2013; Korkmaz et al. 2008; Matsunami et al. 2011). Studies have shown that HBO can increase O_2_ partial pressure and mitochondrial production of H_2_O_2_ in pigeon hearts (Boveris and Chance 1973). Conconi et al. found that ROS production was enhanced by exposure of cultured fibroblasts in vitro to HBO at 2.5 atmosphere absolute (ATA) (Conconi et al. 2003). It is believed that excessive ROS generation, more accurate oxidative stress, and the imbalance between ROS and antioxidant capacity are key factors in the pathological process of stroke (Rahal et al. 2014), especially cerebral ischemia–reperfusion injury (Chan 1996).In recent years, however, the idea that ROS are always detrimental during IS has been challenged. Recent studies have shown that HBO-PC-induced low levels of ROS exert a neuroprotective effect in IS models and stimulate adaptive reactions by enhancing the activity of enzymatic antioxidants and various low molecular weight antioxidants in cells (Benedetti et al. 2004; Conconi et al. 2003), which can remove excess accumulated ROS and protect neurocytes from hypoxic injury. It has also been found that HBO-PC-induced tolerance can be attenuated by the application of antioxidant enzymes (Nie et al. 2006) or oxygen free radical scavengers (Xiong et al. 2001). Guo et al. observed that HBO-PC reduced hemorrhagic transformation via the ROS/Nod-like receptor protein 3 pathway (Guo et al. 2016). These studies suggest that the production of a nonlethal level of ROS might be related to the protective mechanism generated by HBO-PC.

HBO exposure could induce different types of antioxidant enzymes that can protect against ROS, such as SOD (Gregorevic et al. 2001) and CAT (Nie et al. 2006) (Li et al. 2008a).In addition, HBO-PC can reduce MDA levels in the ischemic penumbra and hippocampus. In addition to stimulating antioxidant capacity, ROS in appropriate amounts are important as signaling molecules or messengers in physiological processes (Valko et al. 2007), such as the innate immune response, extracellular matrix dynamics, proliferation, differentiation and cell migration (Montezano and Touyz 2012; Wingler et al. 2011).

#### Nrf2 generation and downstream gene expression

NF-E2-related factor 2 (Nrf2) is a basic leucine zipper transcription factor. A large number of antioxidant proteins are modulated via the mutual effect of the transcription factor Nrf2 with cis-elements in the antioxidant response element (ARE) adjacent to the corresponding gene promoter. After Nrf2 combines with ARE, it constitutes heterodimers with the small musculofascial fibrosarcoma protein that intercede trans-activation. It is important in modulating antioxidant enzyme expression and helping to detoxify and eliminate environmental oxidative stress. In a previous study (Zhai et al. 2016), researchers found that HBO-PC reduced infarct size and alleviated neuronal damage and apoptosis after ischemia in vivo. After repeated HBO exposure, Nrf2 expression, heme oxygenase-1 (HO-1) and GST activities were significantly increased (Zhai et al. 2016). Hyperoxia interference with nuclear factor kappa-B (NF-κB) may mediate the anti-inflammatory state after HBO (De Wolde et al. 2021).

HO-1, which is also called heat shock protein 32 (Hsp32), is a noticeable component of cellular defense enzymes. Previous studies have shown that HO-1 can reduce oxidant-induced tissue damage and ischemia/reperfusion injury (Ahmad et al. 2006; Panahian et al. 1999). Several signaling pathways were reported to be involved in HO-1 expression, such as the phosphatidylinositol-3-kinase (PI3-K)/Akt, mitogen-activated protein kinase (MAPK), antioxidant response element (ARE) in the HO-1 gene promoter, and nuclear factor E2-related factor 2 (Nrf2) pathways. AREs interact with the transcription factor Nrf2 and have been demonstrated to be major regulators of HO-1 transcriptional activation (Alam and Cook 2003). Hyperoxia HBO-PC-associated Nrf2 expression induces the generation of HO-1, which may contribute to its neuroprotective ability (Nesovic Ostojic et al. 2021; Zhai et al. 2016). Under normal conditions, HO-1 is almost undetectable in the brain. It can be rapidly induced in astrocytes and microglia through oxidative stimuli. Although HO-1 is not an antioxidant enzyme, its antioxidant effects are due to its ability to increase antioxidases (Turkseven et al. 2005) and to degrade heme to carbon monoxide, ferrous ions, and biliverdin. HO-1 may act as a novel molecule to protect neurons from acute injury by promoting iron efflux from cells under existing conditions, as both heme and iron are catalysts for free radical generation. Overexpression of HO-1 might also influence the regulation of apoptotic pathway genes such as Bax, Bcl-2, and caspase (CASP) (Vulapalli et al. 2002). In addition, HBO-PC could attenuate CASP levels (Khademi et al. 2020). Therefore, HO-1 expression induced by HBO-PC represents an important neuroprotective mechanism.

GST is an antioxidant enzyme known to protect endothelial cells from damage by oxidants and toxins, which is also regulated by the transcription factor Nrf2 and mediated by oxidative stress levels in cells (Townsend et al. 2009). First, GST expression may rely on multiple signal transduction pathways of ARE, which are related to the indirect effects of oxygen radical intermediates catalyzed through P450 CYP1A1 and the direct action of oxidants and phenolic antioxidants (Hayes and Strange 1995). Second, GSH in cells may enhance GST expression (Bergelson et al. 1994) because ROS production and reduced glutathione consumption may induce the AP-1 complex, resulting in AP-1-mediated transcriptional activation of GST gene expression. Finally, other elements and factors may also modulate GST expression, such as a selenium-related mechanism (Christensen et al. 1994; Moffat et al. 1996). Vnukov et al. observed increased GST levels after conditions of hyperoxia (Vnukov et al. 2017).

### Generation of HIF-1α and expression of EPO

The transcription factor hypoxia-inducible factor-1 (HIF-1) consists of HIF-1α and HIF-1b. It is a critical regulator that induces gene expression and promotes the adjustment and endurance of cells and organisms under hypoxic conditions (Semenza 1998; Wang et al. 1995). Studies have shown that HIF-1α is a critical agent of hypoxia-induced ischemia tolerance (Bruick and McKnight 2001; Choi et al. 2001; Wang et al. 1995) and cross-tolerance (Behrend et al. 2003; Watanabe et al. 2003). In addition to hypoxia, other stimuli, such as vascular hormones, cytokines, growth factors, hyperoxia, viral proteins, and HBO-PC, can also induce HIF-1α. In this respect, HIF-1α seems to act as a universal molecular master switch. HIF-1α targets several critical cellular signaling proteins and enzymes, including EPO, vascular endothelial growth factor (VEGF), glucose transporter 1 (GLUT1), inducible nitric oxide synthase (NOS), and glycolytic enzymes (Chen et al. 2008; Fan et al. 2009; Korkmaz et al. 2008; Semenza 2003a, b; Sharp and Bernaudin 2004), which contribute to cell adaptation to hypoxia. Each of these functions may contribute to the survival of neurocytes. There is growing evidence that HIF-1α is critical for neuroprotection in various models of cerebral ischemia.

It is not hard to understand the production of HIF-1 under hypoxic conditions. However, how can the paradoxical HIF-1 increase be explained under hyperoxic or HBO conditions? Studies suggest that several mechanisms may be involved. First, HBO-PC involves breathing almost 100% oxygen at an air pressure higher than one ATA, which can increase the oxygen content of tissues. When HBO ends, oxygen levels drop to 21% of normal, and brain tissue experiences a state of relative hypoxia. Intermittent exposure to HBO may induce transient hypoxia between HBO exposures, which may induce the expression and/or activity of HIF-1 (Fratantonio et al. 2021; Peng et al. 2008). Second, HBO increased the production of ROS (Benedetti et al. 2004; Conconi et al. 2003; de Wolde et al. 2022; Gregorevic et al. 2001), and increased ROS levels can induce HIF-1 (Guzy et al. 2005; Kietzmann et al. 2001; Peng et al. 2006; Stieg et al. 2022) by downregulating the threshold for HIF-1α and EPO activation. The main ROS species required for HIF-1 induction is H_2_O_2_. Studies have shown that AMP-activated protein kinase (AMPK) is essential for the induction and stabilization of ROS-induced HIF-1α, and restraint of AMPK activity leads to an increase in HIF-1α–pVHL and HIF-1α ubiquitination interactions under H_2_O_2_ (Jung et al. 2008). It is possible that activated AMPK directly phosphorylates HIF-1α and/or VHL, increasing HIF-1α stability by blocking HIF-1a–pVHL interactions and the ubiquitination of HIF-1α to ROS. Moreover, ROS can regulate HIF-1α by activating phosphoinositide 3-kinase/Akt (PI3K/AKT) (Gao et al. 2004), interfering with hydroxylase activity and leading to the accumulation of HIF-1α (Metzen et al. 2003). After blocking the PI3K/Akt pathway with the specific inhibitor wortmannin, HIF-1α and its target gene VEGF protein were significantly inhibited (Li et al. 2008a). Indeed, Gu et al. reported a significant increase in HBO-induced protein expression of HIF-1α and its target gene EPO.

The neuroprotective effect of HIF-1 and its downstream genes has been demonstrated in various studies (Dang et al. 2011; Kietzmann and Gorlach 2005; Sun et al. 2003). To date, over 100 downstream genes of HIF-1 with different functions have been identified (Pan et al. 2021), including vascular endothelial growth factor gene, COX-2 gene, erythropoietin gene, nitric oxide synthase gene, and matrix metalloproteinase gene. EPO is a downstream gene involved in the regulation of HIF-1, which is a hematopoietic growth factor that controls the proliferation, differentiation, and maturation of red blood cells by combining with the surface of erythrocytes and reducing their apoptosis. EPO is also expressed in the central nervous system and exerts effective neuroprotection (Digicaylioglu et al. 1995; Jiang et al. 1996; Morishita et al. 1997). Previous studies have found increased EPO transcription and translation in preconditioned brains (Jones and Bergeron 2001; Prass et al. 2000). Furthermore, exogenous EPO showed neuroprotective effects in vitro (Morishita et al. 1997; Ruscher et al. 2002) and in vivo (Prass et al. 2000; Sakanaka et al. 1998; Sirén et al. 2001). In a study by Gu et al., the author demonstrated that HBO-PC improves functional recovery and diminishes the infarct volume after focal cerebral ischemia (Gu et al. 2008). It was also shown that HBO-PC promoted the activation of HIF-1 DNA binding and increased the mRNA expression of EPO, followed by increased HIF-1 and EPO protein expression in the cortex and hippocampus.

### Induction of heat shock protein

Previous studies have suggested that the protective effect of HBO-PC is related to HSPs (Tsai et al. 2014; Wu et al. 2018). In an animal model of hepatic ischemia–reperfusion injury (Wu et al. 2018), five-day HBO-PC (2.0 ATA, 60 min per session) promoted the expression of HSP70 and reduced hepatic inflammatory and oxidative damage. Intermittent HBO exposure at 1.3 ATA with 20.9% O_2_ was proven to enhance the endurance capacity of well-trained mice by facilitating oxidative and glycolysis capacity with upregulation of HSP70 in skeletal muscles (Suzuki 2019).

HSPs are function-related proteins whose expression increases when cells are exposed to stressful conditions. As early as 1996, Japanese researchers found that repeated HBO-PC induced tolerance against ischemic neuronal injury by inducing HSP72 in the hippocampus (Wada et al. 1996). Subsequent studies suggested that HSP70 overexpression can prevent ischemic damage and protect neurons and glial cells, possibly by preventing protein aggregation, reducing the inflammatory response, refolding incompletely denatured proteins, and inhibiting cell death pathways (Brown 2007). In addition, HBO-PC could increase the expression of other members of the HSP family and exert protective effects in different animal models. Studies included in vivo and in vitro have demonstrated that HBO-PC-induced HSP32 significantly protects spinal cord neurons from oxidative damage and oxygen glucose deprivation injury (Huang et al. 2016). Further investigation found that pretreatment of neurons with the p38 MAPK inhibitor ROS scavenger N-acetyl-L-cysteine or Nrf2 gene knockdown potently reversed HSP32 induction by HBO, which could be enhanced by MEK1/2 inhibitors or gene knockdown. It was suggested that HBO induced the expression of HSP32 via the ROS/p38 MAPK/Nrf2 pathway and that the MEK1/2/Bach1 pathway played a negative role in this process. Another study (Qin et al. 2008) mentioned that ribosomal protein S6 kinase (p70 S6 K) activation after five consecutive HBO-PC sessions may play a role in HSP synthesis and contribute to HBO-PC-induced brain protection in rats. Since p70 S6 K are vital enzymes in protein synthesis, activation of p70 S6 K and an increased ability to synthesize new protein may thus explain the ischemic tolerance of rats after HBO-PC.

### Anti-inflammation and immune modulation

There is increasing evidence that neuroinflammation exacerbates brain damage after cerebral ischemia (Wang et al. 2007). Microglia are said to be the main mediators of the immune responses to multiple injuries and diseases in the central nervous system (Bilimoria and Stevens 2015; Kreutzberg 1996) (Jacobowitz et al. 2012) (Gensel and Zhang 2015). They also determine the effects of inflammation and neuronal function (Valdearcos et al. 2014). Microglia released proinflammatory mediators that caused neuronal dysfunction and cell death (Block and Hong 2005; Lull and Block 2010). Microglia are the major manufacturers of TNF-α in the brain and might play a role in inflammation under certain pathological conditions (Sawada et al. 1989). The regulation of microglial activation might participate in the neuroprotection of HBO-PC, which is shown in intracerebral hemorrhage (Wang et al. 2019) (Yang et al. 2015). However, the role of HBO-PC in IS warrants further exploration.

#### COX-2

Cyclooxygenase-2 (COX-2) catalyzes the formation of arachidonic acid end products, which is a key element of neuroinflammation after ischemia (Candelario-Jalil et al. 2007; Nogawa et al. 1997). Pharmacological blockade of COX-2 with highly alternative inhibitors has a strong neuroprotective effect in experimental animals with focal or global brain ischemia (Nakayama et al. 1998; Sasaki et al. 2004; Xiang et al. 2007). Cheng et al. (Cheng et al. 2011) found that HBO-PC increased viable neurons in cornu ammonis area 1, which was associated with decreased expression of COX-2 in the hippocampus and cortex after ischemia. HBO-PC promoted neurological function and tended to reduce mortality and the number of seizures. However, these positive effects could be eliminated by the application of the COX-2 alternative inhibitor NS-398 prior to HBO-PC. The role of COX-2 in HBO-PC neuroprotection has two facets. On the one hand, HBO-PC could reduce COX-2 upregulation after different neural insults, which has been proven by previous studies. On the other hand, HBO-PC alone has been shown to increase COX-2 protein levels in different CNS disease models, such as surgical brain injury and glucose deprivation injury. This may indicate that HBO-PC protects the cerebrum by promoting COX-2 expression/activation to subinjury. COX-2 inhibitors persistently blocked HBO-PC-induced neuroprotection. Therefore, these inhibitors should be discontinued or replaced with other medications before using HBO-PC.

#### 15d-PGJ2

The cyclopentone prostaglandin 15- deoxy-Δ12,14-PGJ2 (15d-PGJ2) is one of the prostaglandins of the J series (PGJs). 15d-PGJ2 are cyclopentenones synthesized from arachidonic acid through enzymatic transformation by COX and PGD2 synthase following nonenzymatic dehydration synthesis from PGD2 to a variety of PGJs (Saito et al. 2007). It was previously demonstrated that HBO-PC prominently promoted the 15d-PGJ2 level in oxygen-glucose deprivation (OGD)-exposed neurons, a common in vitro model for studying ischemia–reperfusion injury of neuronal cells, which was remarkably blocked by the COX-2 inhibitor NS-398 (Zeng et al. 2012). The results also showed that activation of COX-2 facilitates 15d-PGJ2 production when HBO-PC protects against OGD-exposed neurons. These studies further demonstrated that HBO-PC can guard cultured cortical neurons by COX-2 activation and 15d-PGJ2 release.

Studies have shown that 15d-PGJ2, as an endogenous ligand, has a high affinity for peroxisome proliferator-activated receptors (PPARs) (Forman et al. 1995), which protects the cerebrum and other organs from ischemic insult (Collino et al. 2008; Cuzzocrea et al. 2003; Fong et al. 2010; Takagi et al. 2004). The OGD model is a common in vitro model for studying ischemia–reperfusion injury of neuronal cells. A previous study (Zeng et al. 2012) indicated that OGD exposure caused severe damage to cultured cortical neurons, which was markedly improved by HBO-PC. Furthermore, a study on primary cultured cortical neurons subjected to OGD exposure showed that HBO-PC potently promoted the expression of PPARγ mRNA and protein, PPARγ DNA synthesis activity, and 15d-PGJ2 levels. In addition, the PPARγ antagonist GW9662 notably attenuated the protective effect of HBO-PC in OGD-exposed neurons. 15d-PGJ2 production in OGD-exposed neurons with HBO-PC was blocked by using the COX-2 inhibitor NS-398. These results suggest that HBO-PC can directly protect cultured cortical neurons from OGD damage by activating PPARγ to 15d-PGJ2 production and increasing the activities of downstream antioxidant enzyme.

#### HMGB1

High mobility group box 1 (HMGB1) is well known as a proinflammatory cytokine and is important in the pathogenesis of a variety of diseases, particularly inflammatory diseases (Andersson and Tracey 2011; Sims et al. 2010). HMGB1 can be regulated after transcription, mostly by acetylation of lysine to change its location in the cells (Bonaldi et al. 2003). In a study by Zhao et al., HBO was proven to ameliorate cerebral ischemia injury both in in vivo and in vitro ischemia reperfusion injury models. The mechanism may be that HBO-PC could induce deacetylation of HMGB1 by regulating SIRT1 and inhibiting downstream inflammatory cytokines such as MMP9, TNF-α, IL-6, and IL-1β (Khademi et al. 2020; Wang et al. 2019; Zhao et al. 2021).

### Activation of autophagy

Autophagy is a complicated process in cells that engages in degrading the cell’s own contents via a lysosomal mechanism (Kundu and Thompson 2008). It helps maintain a balance among cell product synthesis, degradation and recycling. Therefore, it is important in cell growth, development, and maintenance of homeostasis. Autophagy is characterized by autophagosome formation. Beclin 1 and LC3 are autophagy-associated proteins that are important in the formation of autophagosomes (Suzuki and Ohsumi 2007). The membrane-bound pathway of LC3 and LC3-II, derived from cleavage of LC3, is an integral part of the autophagosome membrane and is often used in conjunction with the presence of autophagosomes to evaluate autophagy activity (Tanida et al. 2004). Alterations in autophagy activity have been shown to be associated with many diseases, particularly neurodegenerative illnesses, in which inadequate protein clearance might be due to induction or impaired alterations in autophagy-lysosomal degradation pathways (Shacka et al. 2008). Many necrotic apoptotic cells are produced after cerebral ischemia. These necrotic cell products can trigger a local inflammatory response, while degraded intracellular ingredients need to be removed and reused. Since ischemia results in a deficiency of indispensable nutrients, it is reasonable that autophagic pathways activate quickly after ischemia to facilitate cell survival by degrading toxic metabolites (Wang et al. 2010).

In a previous study, Yan et al. revealed that HBO-PC significantly increased LC3-II and Beclin 1 expression and induced the formation of autophagosomes in the ischemic penumbra after cerebral ischemia in rats (Yan et al. 2011). Inhibiting 3-MA autophagy by inhibiting PI3K reversed HBO-PC-induced tolerance to focal cerebral ischemia, while autophagy activated by rapamycin improved subsequent cerebral ischemic damage. The neuroprotective effects of rapamycin appear to be the same as those of HBO-PC. Furthermore, transient focal cerebral ischemia might gently activate autophagy. Cerebral ischemia damage was aggravated by 3-MA therapy. These studies strongly demonstrated that autophagy activation is involved in HBO-PC-induced tolerance facing focal brain ischemia, which is consistent with a formerly reported hypothesis suggesting that HBO-PC protects neurons against brain ischemic insult via stimulating autophagy (Wang et al. 2010). During myocardial ischemia-reperfusion injury, HBO protects cardiac myocytes by reducing inflammation and autophagy(Chen et al. 2017).

It has been shown that ROS produced by mitochondria might be involved in inducing autophagy, causing either cell survival or death, relying on various environments and ROS levels. Current results suggest that ROS also modulate starvation-induced autophagy via the PI3K pathway, which is clearly a survival mechanism (Scherz-Shouval et al. 2007). The neuroprotective effect of HBO-PC was attenuated by applying oxygen radical scavengers(Li et al. 2007; Nie et al. 2006). Increasing evidence has demonstrated that exogenous/endogenous ROS, as significant signaling molecules, participate in autophagy activation and can prevent cells from injury under stress and pathological conditions (Djavaheri-Mergny et al. 2006). It is thus speculated that HBO-PC may induce ROS production and activate autophagy to exert neuroprotective effects against ischemia.

### Anti-apoptosis

Apoptosis refers to programmed cell death and is an essential process for the growth and health of multicellular organisms. HBO-PC may reduce apoptosis in the penumbra by reducing CASP activities (Li et al. 2008b; Petrosillo et al. 2011; Qin et al. 2008), promoting BDNF levels, and inhibiting p38 MAPK activity (Ostrowski et al. 2010). HBO-PC may serve as a powerful prophylactic treatment to seal off the inflammation inherent in stroke and may contribute to the transfer of elastic mitochondria from astrocytes to inflammation-prone neuronal cells to mitigate cell death (Lippert and Borlongan 2019).

#### Cytochrome c (CytoC)

Previous studies have shown that mitochondria are the main regulatory factors in the mechanism underlying preconditioning-triggered endogenous neuroprotection (Correia et al. 2010). During apoptosis, CytoC is released into the cytoplasm from mitochondria and is considered to be a trigger factor for neuronal apoptosis development (Green and Reed 1998). CytoC release activates CASP-3 by activating apoptotic protease activator 1 and procaspase-9 complexes. Activation of CASP-3 leads to cytoskeletal degradation, fragmentation of DNA, and finally cell death. Li et al. found that HBO-PC decreased CytoC release and significantly restrained CASP activity in the hippocampus and penumbra of rats (Li et al. 2009). In their study, Bcl-2 and Bax, two upstream apoptosis factors, were found in mitochondrial pathways. Upregulation of Bcl-2 and an increased Bcl-2/Bax ratio penumbra and hippocampus have been found to attenuate apoptotic cell death.

#### Cystatin C (CysC)

CysC is a cysteine protease inhibitor secreted by 13.3 kDa. It is widely present in the tissues and body fluids of mammals and shows high concentrations in the central nervous system (Håkansson et al. 1996; Turk et al. 2008). Recent investigations demonstrated that CysC could prevent oxidative damage (Olsson et al. 2004). However, a much higher serum CysC level is also thought to be a risk factor for stroke, as well as a predictor for poorer results. Through gene manipulation techniques such as siRNA and constructing CysC knockout rats, the neuroprotective effect induced by HBO-PC was abolished when CysC was knocked out or knocked down (Fang et al. 2017). On the other hand, the use of exogenous CysC in vivo markedly prevented ischemic neuronal injury, suggesting that CysC might be necessary in HBO-PC.

Oxidative stress can trigger lysosomes to breakdown or rupture and release hydrolytic enzymes into the cytoplasm. Several hydrolases, including cathepsins B, L, and D, exist in many neurons. The processes of cell death, including apoptosis and necrosis, occurafter the release of lysosomal histases B and D from the cytoplasm (Repnik and Turk 2010). CysC is primarily distributed in intact lysosomes and is an endogenic protease inhibitor of cathepsins B, H, K, L and S (Gauthier et al. 2011; Watanabe and Forman 2003). In pathological conditions such as apoplexy, cathepsin leakage from the lysosome would destroy both the organelles and the lysosome itself. This process might lead to further release of proteases and exacerbate the damage (Werneburg et al. 2002). Thus, minimizing lysosome damage may be an important mechanism for neural tissue preservation in ischemic reperfusion damage. Fang et al. (Fang et al. 2017) revealed that mannose-binding lectin protein C and CysC had unique variations in mice with less infarction after HBO-PC and brain ischemia. Moreover, he found that CysC is essential for promoting autophagic flux biochemically and morphologically and that HBO-PC and CysC have important translational potential for stroke(Fang et al. 2019). HBO-induced endogenous CysC increase protected lysosomal membrane integrity after apoplexy in wild-type mice but not in CysC siRNA perfusion or CysC−/− mice. In addition, exogenous CysC also has a neuroprotective effect against ischemia-reperfusion damage. The specific role of CysC in HBO-PC needs further investigation in the future.

#### Sirtuin 1 (SIRT1)

SIRT1, a member of the sirtuin family of mammals, is a class III histone deacetylase that produces enzyme activities in the presence of NAD+ (Blander and Guarente 2004). Previous studies indicate that SIRT1 could modulate axon protection and neuronal survival (Gao et al. 2010; Kim et al. 2007) and be critical in neuroprotection (Qiang et al. 2011; Tsai et al. 2015). A previous report demonstrated that SIRT1 regulated the tolerance of HBO-PC-induced ischemia in animal brains (Yan et al. 2013). Yang et al. found that during OGD injury, both HBO-PC and upregulated SIRT1 promoted antiapoptotic Bcl-2 protein expression and reduced proapoptotic cleaved CASP-3 in neurons. Previous studies also showed that HBO-PC increased SIRT1(Fang et al. 2019; Hong-Qiang et al. 2018) and that the Nrf2/antioxidant defense pathway participates in the effects of HBO-PC-induced long-lasting neuroprotection of SIRT1 against transient focal cerebral ischemia (Xue et al. 2016). In contrast, the enhanced SIRT1 inhibition induced by HBO-PC inhibited Bcl-2 and increased cleaved CASP-3 protein, indicating that apoptosis mediated by SIRT1 is involved in HBO-PC neuroprotection. Multiple findings have shown that SIRT1 deacetylates p53, which is also an apoptosis-inducing protein. The deacetylation of p53 by SIRT1 results in apoptosis inhibition (Alcendor et al. 2004). Thus, it is conceivable that SIRT1 may be involved in the neuroprotection of HBO-PC by regulating apoptosis through deacetylation of p53.

### Brain-derived neurotrophic factor (BDNF)

Neutrophins are candidate genes for HBO-PC neuroprotection. A previous study reported that the level of BDNF protein decreased in transient forebrain ischemia (Kokaia et al. 1996). However, in models treated with HBO-PC, BDNF expression increased in the cerebral cortex and CA1 early after global ischemia (Chavko et al. 2002). The protein expression of p75NTR (a low-affinity receptor for BDNF) was also significantly increased within 12–24 h after HBO treatment. NF-κB may be included in the upstream pathway of BDNF upregulation, where the BDNF gene is located in a promoter 3 region (Marini et al. 2004). Thus, upregulation of NF-κB by hyperoxia or HBO may induce BDNF, particularly in the “eternal” five HBO regimen (Tähepõld et al. 2003).

The effects of BDNF downstream might include the inhibition of p38/MAPK activity through the suppression of p38/MAPK phosphorylation (Yamagishi et al. 2003). Recent investigations indicated that the p38 MAPK signaling pathway might serve as a rapid reaction signal because it is triggered in flimsy neurons within a few minutes after global brain ischemia (Sugino et al. 2000; Takagi et al. 2000) and plays a crucial role in ischemic or cross tolerance (Nishimura et al. 2003; Ostrowski et al. 2008). Studies have shown that P38/MAPK inhibition contributes to cell survival in focal and global brain ischemia (Barone et al. 2001; Sugino et al. 2000), and pretreatment with SB203580 reduced ischemic- (cross-) tolerance (Nishimura et al. 2003; Sun et al. 2006; Zheng and Zuo 2004). HBO-PC inhibited p-p38 expression after ischemia, suggesting that HBO-PC may induce ischemic tolerance by enhancing BDNF and inhibiting P38 activation during reperfusion (Ostrowski et al. 2008).

### Blood-brain barrier and MMP-9

In the subacute phase of IS, neuroinflammation occurs due to the release of cytokines, chemokines, and matrix metalloproteinases (MMPs) (Fu et al. 2022). The overexpression of MMPs can increase the permeability of the blood-brain barrier (BBB), cause migration waves of white blood cells into the infarct area, and aggravate inflammatory activities. A variety of cell phenotypes containing neurovascular units in the penumbra are also susceptible to the pathological mechanisms mentioned above (Wang et al. 2021a).

#### Blood–brain barrier (BBB)

The BBB consists of tight junctions (TJs) among the capillary basal layer, endothelial cells, pericytes and astrocyte endfeet and is a highly alternative penetration barrier (Ballabh et al. 2004). It is crucial in maintaining cerebral homeostasis. BBB breakdown causes edema or hemorrhage of angiogenesis and neuronal cell death, which may cause the pathophysiology of ischemic brain injury (Lee et al. 2013). The complicated mutual effects of cytoskeletal proteins and tight junction proteins (TJPs) formed TJs between cerebral endothelial cells. TJs include the interactions of zonula occludens (ZO), occluding, claudins, and cingulin (Wolburg and Lippoldt 2002). TJPs promote endothelial electrical resistance and reduce paracellular permeability (Coisne and Engelhardt 2011). Changes in TJP expression and distribution can cause the loss of BBB integrity and BBB breakdown (Ballabh et al. 2004; Luh et al. 2010).

Complex TJPs regulate the opening of TJs, such as peripheral membrane protein family members, transmembrane proteins, and adhesion molecules (Farkas et al. 2012), while occlusion modulates their sealing. Blocking occluding expression alone is sufficient to induce dysfunction of TJs (Persidsky et al. 2006; Tavelin et al. 2003). ZO-1 is a bridge between transmembrane proteins and skeleton proteins, which plays a key role in TJP stability and function (Abbott et al. 2006; Xia et al. 2013). In the early phase of hypoxia-ischemia, the BBB loses permeability barrier function, which may involve endothelial TJ dysfunction, relocation and dysregulation of occludin and ZO-1 (Mark and Davis 2002; Witt et al. 2003). In a previous study (Hao et al. 2016), hypoxia induced the downregulation of occludin expression and ZO-1 relocation from the membrane to the cytoplasm, which could be reversed by HBO-PC before hypoxia. The author believed that this may be the reason why TEER in the HBO-PC group was higher than that in the hypoxia group. Chen et al. found that hyperoxia could reduce damage to the BBB, attenuate brain cell edema, lower intracranial pressure, improve cerebral blood flow, especially around areas of brain damage, and activate neurons in hemispheric areas(Chen et al. 2020). These results demonstrated that HBO has the potential to protect the integrity of the BBB but need to be further validated.

#### MMP-9: extracellular matrix protein laminin degradation

During the last decade, accumulated data from clinical and experimental studies have confirmed that MMP-9 plays a critical and harmful role in IS and reperfusion injury (Zalewska et al. 2003). Numerous studies have detected a marked increase in MMP-9 expression after IS, which is related to various complications, such as excitotoxicity, neuronal damage (Lee et al. 2004b), apoptosis (Copin et al. 2005; Lee and Lo 2004a), oxidative stress (Kelly et al. 2008), and disturbance with oxidative repair of DNA (Yang et al. 2010). Most importantly, BBB breakdown causes brain edema and hemorrhagic transformation (Zhao et al. 2006). In focal brain ischemia models, MMP-9 KO mice showed noteworthy BBB protection (Asahi et al. 2001).

A recent study proved that HBO-PC reduced the activity and tissue expression of brain MMP-9, and improved cell death after global brain ischemia (Ostrowski et al. 2010). Interestingly, the study found that HBO-PC improved the tissue expression of MMP-9, suggesting that the HBO-PC mechanism may deplete brain MMP-9 storage. This exhaustion would reduce MMP-9 after brain ischemia (Lalu et al. 2002). Previous studies found that HBO-PC modified neurological deficits and reduced hemorrhage; MMP-2 and MMP-9 activities were also reduced (Soejima et al. 2013; Wang et al. 2019). In these cases, the decrease in MMP-9 activity in the HBO-PC group would simply reflect MMP-9 storage depletion in the cerebrum, although other preconditioning mechanisms should also be taken into consideration. In addition, tissue inhibitor of metalloproteinases-1 (TIMP-1) is the target gene of HIF-1 and can inconvertibly inactivate MMP-9 (Gomez et al. 1997). It is necessary to further investigate whether HBO-PC can induce TIMP-1 and then block MMP-9 to improve cell death after global cerebral ischemia.

### Stem cells

Stem cells (SCs) are positioned in mature brain niches with protective and recuperative functions by migrating to the damaged area(Sullivan et al. 2015). There are two main types of stem cells based on the developmental stage: embryonic SCs (ESCs), which are separated from the inner cell mass of the blastocyst, and adult SCs (ASCs), which can be identified in diverse adult tissues, involving epidermal SCs, mesenchymal SCs (MSCs), hematopoietic SCs (HSCs), and neural SCs (NSCs)(Gao et al. 2018). Neural stem cell therapy has been shown to achieve promising therapeutic effects in IS through two strategies: transplantation of exogenous NSCs and promotion of self-repair of endogenous NSCs (Huang and Zhang 2019). HBO has a dynamic effect on a diverse stem cell population in vivo. Strikingly, HBO may increase total accessible endogenous stem cells by raising many circulating stem cells in a pressure-susceptive manner(Zhang et al. 2021) and by upregulating the proliferation of NSCs within their neurogenic niches in the adult brain. The mechanism of HBO in NSC proliferation might involve the upregulation of crucial regulatory molecules, such as its receptor (VEGFR2), vascular endothelial growth factor (VEGF) (HIF-1α downstream target), ERK, and CREB(Liska et al. 2018). HBO boosts the differentiation of NSCs into oligodendrocytes and neurons and decreases astrocytes in vitro, probably through modulating the Wnt3/nuclear b-catenin and BMP2 signaling pathways (Chen et al. 2019).

Delayed grafting and failed engraftment are the main barriers to successful umbilical cord blood (UCB) transplantation (Aljitawi et al. 2014). HBOT can be combined with stem transplantation to improve graft survival and assuage the inflammatory response (Geng et al. 2015). Animal studies found that umbilical cord MSC transplantation combined with HBOT had significantly superior therapeutic outcomes over monotherapy in the treatment of traumatic brain injury (Zhou et al. 2016), hypoxic-ischemic brain damage(Ma et al. 2015), and spinal cord injury (Geng et al. 2015). Heyboer et al. found that HBO-PC can be performed simultaneously with stem cell preconditioning, which can provide an effective dual treatment method for patients at high risk of apoplexy, using preemptive neuroprotective methods and post hoc neurorestorative biologics (Liska et al. 2018). HBO-PC promoted homing of transplanted UCB hematopoietic stem/progenitor cells (HSPCs) to the bone marrow(Cheung et al. 2018) by reducing systemic EPO levels in the recipient (Aljitawi et al. 2016). In addition, HBO-PC improved myeloid, B cell, and T-cell engraftment in a murine transplant model (Aljitawi et al. 2014). The combination of HBO-PC and stem cell therapy has been successfully used in cutaneous flaps (Tenenhaus et al. 2018) and traumatic brain injury(Zhou et al. 2016).

Transplantation of umbilical cord mesenchymal stem cells combined with HBO treatment is superior to monotherapy in repairing traumatic brain injury and can enhance the recovery of neurological function. A clinical study showed that HBO-PC decreased the period to neutrophil and platelet implantation and decreased the application of granulocyte colony-stimulating factor. Heyboer et al. (Heyboer et al. 2014) found that HBO at 2.5 ATA fostered more stem/progenitor cell mobilization than at 2.0 ATA.

In summary, HBO-PC might be considered a safe and effective method to combine with SCs therapy to prevent IS.

## Concerns about HBO-PC

Almost all research on HBO-PC was conducted in animal models or healthy volunteers. In clinical application, HBO-PC is mainly used in high-risk populations with neurological diseases. Therefore, the applicability of the above results needs re-evaluation. It is still unclear whether HBO-PC is cost-effective, which is a critical issue in almost all preventive applications and a defining factor in HBO-PC. It requires good patient compliance, as it usually takes approximately one to two hours and several episodes. In addition, although the benefits of HBO-PC on IS have been widely found in different models, HBO-PC is still controversial in terms of treatment duration, stress parameters, number of sessions, and species (Prass et al. 2000; Wang et al. 2019, 2021; Zhang et al. 2004).

### HBO-PC sessions

The optimal doses of HBO remain unclear. Ischemic tolerance is negated when air (20% oxygen) rather than 100% O2 is applied during preconditioning, indicating that 100% O_2_ is required in the HBO-PC mechanism (Wada et al. 2001). It was previously believed that a single HBO treatment was not enough to induce tolerance to ischemia in the cerebrum. It was recommended that more sessions of HBO-PC (e.g., five times) should be considered, as it was more valid than three-day HBO-PC (Ostrowski et al. 2008). Nevertheless, one study suggested that the neuroprotective effects of a single HBO session in newborn rats might be similar to those of hypoxia pretreatment (Freiberger et al. 2006). Another study found that HIF-1α protein levels rose more significantly after three days (six HBO episodes) (Peng et al. 2008). Long-term preconditioning within 24 h before injury (2.5 ATA for 60 min per day for five consecutive days) effectively established tolerance to transient focal ischemia with hemorrhagic transformation, global brain ischemia, and surgical brain injuries in rats (Jadhav et al. 2010; Ostrowski et al. 2008b; Soejima et al. 2012). Therefore, while short-term pretreatments may be effective, longer pretreatments, such as three to five days, should be considered to attain more valid protection.

### HBO-PC pressure

HBO at high pressure is known to cause several complications, such as seizures (Chavko et al. 2001). Preconditioning with three-ATA-HBO has been shown to be safe and most effective in a rat model of forebrain ischemia without causing seizures (Hirata et al. 2007; Yamashita et al. 2009). Typically, the HBO-PC is conducted at two to three ATA. Each session lasts 60 to 90 min. However, recent studies on different diseases showed that lower pressure HBO at one to two ATA might also exert protective effects as higher pressure. Therefore, further investigation about the optimal protocol of HBO-PC is needed.

### HBO-PC timing

Another issue is the timing between the last HBO exposure and subsequent insult. A 24-hour interval is most commonly used in HBO-PC. Hirata et al. observed the time window of HBO-PC confronting ischemic damage and determined that the neuroprotective effect occurred at six, 12, and 24 h, rather than 72 h, following the last preconditioning (Hirata et al. 2007). However, another study showed that acute pretreatment (three HBO treatments of 60 min at 2.5 ATA occurring within 24 h before MCAO) was less valid when neurological dysfunctions were evaluated 24 h after MCAO (Ostrowski et al. 2008b). In the same way, a protective effect was found two days following ischemia (Li et al. 2009b). How long the neuroprotective effect of HBO-PC lasts remains to be tested in the future.

In conclusion, HBO-PC might be an effective method to prevent IS, and its mechanisms of action may involve the induction of antioxidant activity, HIF-1α, HSP, and autophagy, inhibition of apoptosis, and inflammation. However, it remains unclear whether the target regimen of preconditioning varies from person to person. Before HBO-PC is widely used in IS, the optimal pretreatment scheme should be determined.

## Data Availability

Data sharing is not applicable to this article as no datasets were generated or analyzed during the current study.
